# Erfolgreiche Behandlung eines Patienten mit unipolarem depressivem 48-h-Ultrarapid-Cycling mit Lithium

**DOI:** 10.1007/s00115-026-01952-9

**Published:** 2026-02-23

**Authors:** Georg Juckel, Kai Wetzel, Paraskevi Mavrogiorgou

**Affiliations:** Klinik für Psychiatrie, Psychotherapie und Präventivmedizin, LWL-Universitätsklinikum der Ruhr-Universität Bochum, Alexandrinenstr. 1, 44791 Bochum, Deutschland

## Hintergrund

Im Jahre 2000 publizierten wir den Fall eines bipolaren 48-h-Ultrarapid-Cyclings mit ausgeprägten depressiven und manischen Phasen, die wir sowohl objektiv psychopathologisch als auch vielfältig neurobiologisch nachweisen konnten [1]. Der 67-jährige männliche Patient konnte dann erfolgreich mit Valproat behandelt werden; weitere psychische oder biologische Schwankungen waren nicht mehr erhebbar. Während das Rapid-Cycling durch das Auftreten von mindestens 4 depressiven und/oder manischen Episoden oder 2 vollständigen bipolaren Zyklen pro Jahr definiert ist, ist das Ultrarapid-Cycling gekennzeichnet durch unipolare oder bipolare Zyklen, die 48 h andauern, d. h. einen konstanten Rhythmus von 24 h depressivem Zustand gefolgt von 24 h euthymen oder manischem Zustand aufweisen.

Im Vergleich zum Rapid-Cycling ist das Ultrarapid-Cycling extrem selten. Gelenberg et al. [2] zählten in der Literatur von 1804 bis 1978 lediglich 8 bipolare und 4 unipolare Fälle von ultrarapiden Zyklen. Bis zum Jahre 2000 wurden unseres Wissens nur 8 weitere Fälle gemeldet und bis heute sind nur eine Handvoll zu unserem damaligen Fall hinzugekommen. Die meisten dieser Patienten sind männlich und über 60 Jahre [3], sodass immer wieder eine Debatte über eine hirnorganische Entstehung geführt wurde. Solche Patienten berichten häufig eine ausgeprägte positive Familienanamnese psychiatrischer Erkrankungen [4].

Die Behandlung des depressiven Ultrarapid-Cyclings ist nicht ganz einfach und das Ansprechen auf die therapeutischen Strategien ist im Allgemeinen eher schwer zu prognostizieren. Schlafentzug in Kombination mit einer Behandlung mit Tranylcypromin war in einem Fall mit unipolarem Ultrarapid-Cycling erfolgreich [5]. Amitriptylin verringerte die Amplitude der depressiven Stimmung in einem Fall mit unipolaren ultrarapiden Zyklen [2]. Während Carbamazepin bei unipolaren und bipolaren Fällen ultrarapider Zyklen nur zu einer teilweisen Besserung führte, sprachen diese Patienten gut auf Lithium an [6], während es bekanntlich beim einfachen Rapid-Cycling eher unwirksam sein dürfte. Dies ist aufgrund der wenigen Fälle sicherlich nicht generalisierbar und aufgrund der Seltenheit der Fälle ist das Phänomen des Ultrarapid-Cyclings bis heute wenig erforscht. Die Fälle, über die berichtet wurde, wurden meist im akuten Zustand untersucht.

Wir hatten unseren damaligen Patienten mit bipolarem 48-h-Ultrarapid-Cycling [1] vor und während der erfolgreichen Behandlung klinisch und neurobiologisch untersucht. Vor allem die rhythmischen Anstiege von Kortisol und Wachstumhormon (HGH) und die Erniedrigung von Metanephrin und Norepinephrin waren charakteristisch für die depressiven Tage. Daher haben wir bei dem jetzigen Patienten mit einem unipolar depressiven Ultrarapid-Cycling neben dem naheliegenden Serotonin, das wir elektrophysiologisch mittels LDAEP bestimmt haben, primär diese Parameter untersucht und ihn therapeutisch auf Lithium mit einem ausreichenden Wirkspiegel (zuletzt 0,63 mmol/l) und ohne erkennbare UAW eingestellt.

## Fallbeschreibung

### Anamnese

Der 73-jährige männliche Patient, der schriftlich sein vollumfängliches Einverständnis zur wissenschaftlichen Veröffentlichung seines Falls gegeben hat, kam über eine Selbsthilfegruppe für depressiv erkrankte Menschen aus dem westlichen Ruhrgebiet zu uns an das LWL Universitätsklinikum Bochum, Klinik für Psychiatrie, Psychotherapie und Präventivmedizin. Seine Krankheitsgeschichte mit einer primär affektiv-depressiven Erkrankung begann 2017, also mit 67 Jahren. Zu dieser Zeit kehrte er nach 3 Jahren aus Kanada von seiner letzten beruflichen Station zurück. Grund war die Vorbereitung in Deutschland auf einen weiteren unbefristeten Aufenthalt in Kanada. Der Tod seiner Mutter im Januar 2018 und die fortschreitende depressive Erkrankung führten dann aber dazu, dass sich Kanada in der weiteren Lebensplanung nicht mehr realisieren ließ: Ende 2019 stationäre psychiatrische Behandlung in Krefeld mit der Diagnose einer Depression; anschließend in dortiger ambulanter Behandlung mit diversen Antidepressiva jedoch ohne größeren Erfolg; seit über 2 Jahren jedoch nicht mehr, nur noch in hausärztlicher Behandlung, obwohl fortgesetzte und zunehmende Symptome im Sinne eines zunehmenden unipolaren Ultrarapid-Cyclings, gesichert seit min. Anfang 2024 also ein knappes Jahr vor Aufnahme in unserer Klinik. Seit Herbst 2024 kam es nochmals zu einer deutlichen Verschlechterung der jeweils depressiven Tage bis hin zu akuter Suizidalität und noch stärkerer Akzentuierung eines ausgestanzten 48-h-Ultrarapid-Cycling-Rhythmus.

### Diagnostik

Nach ambulanten Kontakten zur ersten psychiatrischen Einschätzung wird der Patient für eine Woche Anfang November 2024 zur engmaschigen klinischen und apparativen Untersuchung aufgenommen. Es finden sich objektivierbar ein 48-h-Rhythmus mit einem Tag mindesten mittelschwerer, wenn nicht schwerer Depression und einem folgenden Tag der euthymen Beschwerdefreiheit. Aufgrund der Tatsache, dass viele Patienten mit einem Ultrarapid-Cycling eher älter sind, kam zur evtl. differenzialdiagnostischen Einschätzung der Frage der hirnorganischen Abklärung eine besonders Wichtigkeit zu. So wurde der Patient im Vorfeld der Aufnahme ausführlich bez. Labor, EKG, EEG, 3‑T-MRT und Neuropsychologie untersucht. Neuropsychologisch wurde mittels TMT‑A, TMT‑B und MMST gefunden, dass im Screening keine Probleme von Gedächtnis, Konzentration und Aufmerksamkeit zu eruieren sind und die kognitiven Leistungsfähigkeiten sich eher im oberen durchschnittlichen Normbereich befinden. Im EEG findet sich ein Normalbefund mit einem regelmäßigen und gut ausgeprägten α‑EEG mittlerer Amplitude und einer eher höheren Frequenz mit ein wenig β‑Einlagerung, aber mit keinen Zeichen für einen Herd oder gesteigerter hirnelektrischer Erregbarkeit. Auch im MRT wurden altersentsprechende Normalbefunde erhoben: keine Hinweise auf Hirnatrophie, kein akutes oder entzündliches Geschehen, keine Raumforderung, „noch moderat ausgeprägte“ mikroangiopathische Marklagergliosen mit Beteiligung der Balkenstrahlung. Alle weiteren allgemeinen laborchemischen Parameter waren im Normbereich, das EKG unauffällig (Sinusrhythmus, HF 70/min, Indifferenztyp). Die orientierende internistische und neurologische körperliche Untersuchung blieb ohne Befund.

### Zusatzdiagnostik

Im Verlauf des stationären Aufenthalts, bei dem Schlafverhalten und tägliche Aktivitäten nahezu konstant waren, führten wir tägliche Blutentnahmen morgens zwischen 8 und 10.00 Uhr und die Abgabe von 24-h-Sammelurin durch, um so weitere Parameter zur Vervollständigung einer Diagnostik der Verdachtsdiagnose eines Ultrarapid-Cyclings zu stellen. Es erfolgte zudem täglich Psychometrie mit Tests zur Erfassung depressiver Symptomatik sowie einer visuellen Analogskala. Anhand neurobiologischer Befunde konnte der Wechsel in der Psychopathologie mit einem euthymen, gefolgt von einem z. T. schwer depressiven Tag nachvollzogen werden. Es wurde Laborchemie (Blut, Sammelurin) sowie EEG (LDAEP als Indikator der serotonergen Neurotransmission, [7]) ausgewertet: Neben der Psychopathologie gemessen mit BDI und VAS (BDI euth./dep: 5, 17, 5, 17, 5, 17; VAS Stimmung: 10., 0, 8. 1, 9, 1; VAS Antrieb: 10, 2, 9, 2, 9, 2) fanden sich systematische Schwankungen insbesondere im Sammelurin bez. der Monamine Noradrenalin, Serotonin und Dopamin (euth./dep. 446, 182, 330, 194, 194, 107 ug/l). Am depressiven Tag war die LDAEP gegenüber dem darauffolgenden euthymen Tag stärker im Sinne einer reduzierten serotonergen Neurotransmission (0,084 vs. 0,044 µV/10 dB). Vor diesem Hintergrund eines uhrartigen Verhaltens von klinischen und neurobiologischen Befunden mit 24 h Depression gefolgt von 24 h Euthymie wurde von uns die Diagnose gestellt: rezidivierende depressive Störung, gegenwärtig mittelgradige Episode (ICD-10: F33.1) mit phänomenologisch und biologisch gezeigtem 48-h-Ultrarapid-Cycling.

### Behandlung und Verlauf

Der Patient wird anschließend auf Lithium (Quilonum ret. 1‑0-1) eingestellt bei stets suffizientem Blutplasmaspiegel > 0,6 mmol/l. Es traten keinen nennenswerten UAW auf. Der Patient respondierte prompt darauf mit gänzlicher subjektiver und objektiver Symptomfreiheit, d. h. er gibt an, seitdem keine depressiven Tage mehr zu erleben, und auch psychopathologisch objektiv fanden sich seit ca. 12 Monaten keine depressiven Symptome mehr. Es gibt auch des Weiteren keine Hinweise auf das Schwanken entsprechender neurobiologischer Parameter. Gesundheitliche Stabilität und Lebensqualität des Patienten sind vollumfänglich zurückgekehrt, wofür er sich auch angesichts seines langen Leidens mit oftmals quälenden Suizidgedanken, die er primär wegen seiner Familie nicht entsprechend umsetzte, sehr dankbar zeigt.

## Diskussion

Im Gegensatz zu unserem früheren Fall und der Literatur mit den wenigen Fallberichten zu meist bipolarem Ultrarapid-Cycling, auf die wir uns hier im Wesentlichen beziehen, haben wir hier einen der seltenen Fälle eines Patienten mit einem unipolaren Ultrarapid-Cycling (1 Tag depressive, 1 Tag euthym) beginnend mit einem eher unspezifischen depressiven Beschwerdebild und dann seit einem Jahr mit eindeutigem 48-h-Rhythmus beschrieben. Neben den subjektiven Schilderungen des Patienten, den klinischen Eindrücken und der psychopathologischen Befunderhebung konnte dies psychometrisch objektiviert und mithilfe neurochemischer und neurophysiologischer Befunde erhärtet und gestützt werden. Unser Patient zeigte die allgemeinen klinischen Merkmale vergleichbar anderer Ultrarapid-Cycling-Patienten: männlich, älter, später Beginn, rasche Ausprägung des Symptombildes und positive Familiengeschichte für affektive Störungen. Es wurde keine andere Ursache für dieses Krankheitsbild wie hirnorganische Prozesse, Suchterkrankung oder Persönlichkeitsstörung gefunden.

Im Gegensatz zum einfachen Rapid-Cycling konnte hier erfolgreich eine Behandlung monotherapeutisch allein mit Lithium bei hoher Verträglichkeit und Fehlen jeglicher UAW durchgeführt werden, sodass sich Rhythmik und Symptomatik nahezu komplett zurückgebildet haben, was auch für die neurobiologischen Parameter gelten dürfte. Auffällig sind unsere insgesamt konsistenten Befunde zum monaminergen System mit Schwankungen von Tag zu Tag passend zur depressiven Psychopathologie bez. Dopamin, Noradrenalin und Serotonin. Auch unser langjährig erforschter Indikator für das zentralnervöse synaptisch ausgeschüttete Serotonin, nämlich die Lautstärkeabhängigkeit akustisch evozierter Potenziale [7], wies zwischen depressiven und euthymen Tag bei unserem Patienten eine entsprechende Veränderung auf, passend zu den Befunden bei Patienten mit anderen depressiv-affektiven Erkrankungen.

Jedoch wiesen nicht alle gemessenen biologischen Parameter eindeutige stimmungsabhängige rhythmische Schwankungen auf, was z. T. den Befunden in der Literatur entspricht. So zeigten im Gegensatz zu unserem damaligen bipolaren Ultrarapid-Cycling-Fall [1] die Steroidhormone bei dem jetzigen Patienten keine eindeutigen Auffälligkeiten. Schilddrüsenhormone waren damals wie jetzt stabil und zeigten keine Änderungen in Beziehung zu den rhythmischen Schwankungen der Psychopathologie.

Die neuere Literatur zum Ultrarapid-Cycling ist überschaubar. So fanden Ulke et al. [8] aus Leipzig, dass sich bei einem neuerkrankten 46 Jahre alten Mann mit bipolarer Störung „EEG arousal states“ periodisch änderten in den phasenrhythmisch alle 24–72 h auftretenden hypomanen Zuständen, während diese in den Tagen der Depression stabil blieben. Dieser Patient sprach zunächst gut auf die Kombination von Quetiapin bis 300 mg plus Lamotrigin an. Später musste dies ersetzt werden durch Valproat 2000 mg sowie Lithium, Quetiapin und L‑Thyroxin, was die endgültige Stabilisation erbrachte. Auch das EEG war dann nicht mehr auffällig. Wilk und Hegerl [9] berichteten in einer Übersicht, dass die Tageszeit des Umschlags von Stimmung und Antrieb im Sinne eines Phasenwechselns bei 48-h-Ultrarapid-Cycling immer in den Nachtstunden stattfinden würde. Dies wird in dem guten Überblick über Ultrarapid-Cycling von bipolaren Störungen durch Goldberg [10] bestätigt. Dabei ist es noch ungeklärt, ob Rapid-Cycling überhaupt eher einen State- oder Trait-Zustand bei affektiven Störungen darstellt, und auch sind Ultrarapid-Cycling und ultradianes Rapid-Cycling von einer gemischten Episode und Zyklothymie schwierig abzugrenzen. Auffällig ist, dass Ultrarapid-Cycling-Fälle, insbesondere die mit dem 48-h-Rhythmus, häufig bei älteren Patienten, so wie bei unserem hier berichteten Patienten, auftreten, sodass die Vermutung naheliegt, dass evtl. eine hirnorganische Genese oder Mitverursachung diskutiert werden muss. Dazu passt, dass eine EKT-Behandlung evtl. auch kognitive Störungen induzieren kann und damit eine leichte hirnorganische Symptomatik. Hierdurch könnte die Gefahr eines Ultrarapid-Cycling wachsen, ähnlich wie dies bez. der durch Lithium hervorgerufenen kognitiven Einschränkungen beschrieben ist. So berichten Zavorotny et al. [11] von einem Ultrarapid-Cycling-Fall nach EKT. Eine 66 Jahre alte Frau mit einer seit 35 Jahren bestehenden bipolaren Störung erhielt wegen einer starken bipolaren Depression eine EKT-Serie und entwickelte darunter starke tagesbezogene Stimmungsschwankungen, die erfolgreich mit Lithium behandelt werden konnten. Moslov et al. [12] berichten über den Zusammenhang von EKT und bipolarem Ultrarapid-Cycling. Eine EKT mit 5 bis 10 Sitzungen sei als Methode wirksam bei Rapid-, aber auch bei Ultrarapid-Cycling-Fällen. Die Aufhebung des Cyclings würde damit in nahezu der Hälfte aller Fälle gelingen. Bürgy [13] berichtete von einer 19 Jahre alten Frau mit einem ultradianen Rapid-Cycling mit einem bipolaren Verlaufstyp (nachts depressive, tagsüber manische Symptomatik). Er konnte sie erfolgreich behandeln mit der Kombination von Lamotrigin und Quetiapin. Einen weiterhin gültigen Überblick über ultradianes bipolares Rapid-Cycling geben Kramlinger und Post [14] vor allem hinsichtlich der jeweiligen Phasenlängen bei diesen Störungsbildern.
